# Recent Advances in Endothelial Colony Forming Cells Toward Their Use in Clinical Translation

**DOI:** 10.3389/fmed.2018.00295

**Published:** 2018-10-23

**Authors:** Koralia E. Paschalaki, Anna M. Randi

**Affiliations:** Vascular Sciences, National Heart and Lung Institute, Faculty of Medicine, Imperial College London, London, United Kingdom

**Keywords:** endothelial progenitors, blood outgrowth endothelial cells, vascular regeneration, cell therapy, gene therapy, tissue bioengineering

## Abstract

The term “Endothelial progenitor cell” (EPC) has been used to describe multiple cell populations that express endothelial surface makers and promote vascularisation. However, the only population that has all the characteristics of a real “EPC” is the Endothelial Colony Forming Cells (ECFC). ECFC possess clonal proliferative potential, display endothelial and not myeloid cell surface markers, and exhibit pronounced postnatal vascularisation ability *in vivo*. ECFC have been used to investigate endothelial molecular dysfunction in several diseases, as they give access to endothelial cells from patients in a non-invasive way. ECFC also represent a promising tool for revascularization of damaged tissue. Here we review the translational applications of ECFC research. We discuss studies which have used ECFC to investigate molecular endothelial abnormalities in several diseases and review the evidence supporting the use of ECFC for autologous cell therapy, gene therapy and tissue regeneration. Finally, we discuss ways to improve the therapeutic efficacy of ECFC in clinical applications, as well as the challenges that must be overcome to use ECFC in clinical trials for regenerative approaches.

## Introduction

The search for endothelial progenitors started in the late 1990s, when Asahara et al. reported the existence of circulating cells with endothelial surface markers and repair capacity, and labeled them “endothelial progenitors” (1). Shortly after, Lin et al. reported the presence of circulating cells which could differentiate into cells with the phenotypic characteristics of vascular endothelium (2). These studies triggered what was to become a decade-long debate on the existence and nature of “endothelial progenitor cells.” The controversy sadly damaged the “brand” EPC, so that the acronym is no longer in favor. EPC have been defined as circulating cells with the ability to differentiate into mature endothelial cells and contribute to postnatal vasculogenesis and endothelial repair at sites of vascular damage (3). We now know that some of the populations originally defined as EPC do not fulfill this definition. These include myeloid angiogenic cells (MAC), also called circulating angiogenic cells (CAC) or “early” EPC, which in fact are of hematopoietic origin and promote angiogenesis through paracrine mechanisms, but cannot give rise to mature endothelial cells (4–8). The population of circulating cells, which show clonal potential, ability to give rise to mature endothelial cells and promote vascular formation *in vitro* and *in vivo*, have been called endothelial colony forming cells (ECFC), blood outgrowth endothelial cells (BOEC), or late outgrowth EPC (4). For years, different groups have used their favorite acronym; it was recently suggested that the term “ECFC” should be used to harmonize the literature (4). ECFC are more frequently isolated from cord blood or peripheral blood (2, 9) but can also be isolated from tissue-resident vascular endothelium, including human umbilical cord (9, 10), pulmonary artery endothelium (11), lung tissue (12), placenta (13), or can be derived from induced pluripotent stem cells (iPSC) (14).

**Figure 1:**
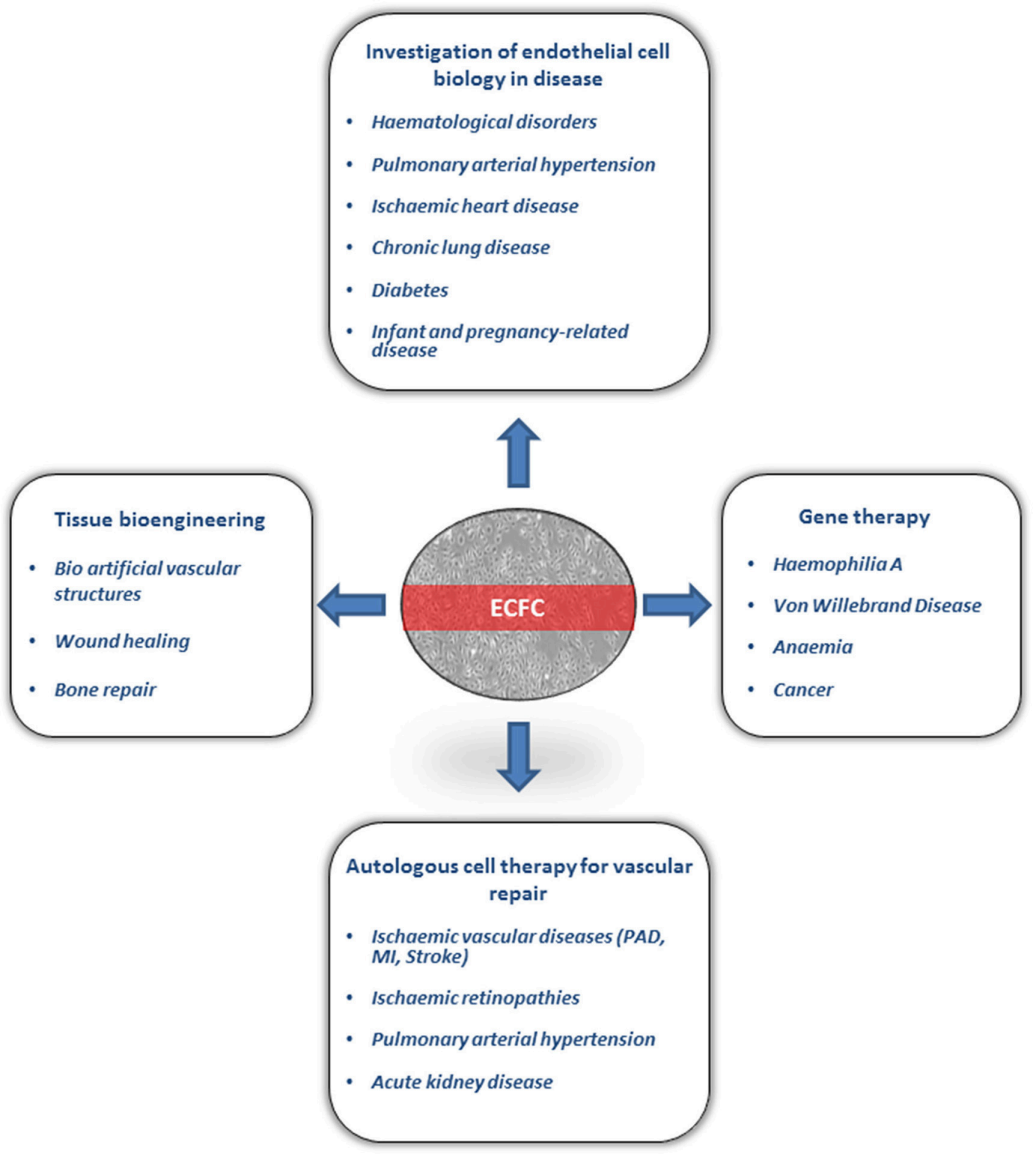
Clinical applicaions of Endothelial Colony Forming Cells (ECFC). ECFC have been used for investigation of endothelial cell biology in several diseases. ECFC are currently under investigation in preclinical models for autologous cell therapy, gene therapy and tissue bioengineering. PAD, peripheral arterial disease; MI, myocardial infraction.

The origin of circulating ECFC still remains unclear. The first study from Lin et al. on ECFC from circulating blood of patients undergoing bone marrow transplantation suggested a bone-marrow origin (2). Subsequent studies were unable to confirm these findings and proposed that ECFC are more likely to originate from tissue vascular niches (15). This is in line with recent findings suggesting the existence of vascular resident endothelial cells with clonal potential that contribute to postnatal angiogenesis *in vivo*, which is one of the main characteristics of ECFC (16–18). Transcriptome analysis of peripheral blood ECFC samples suggests a profile close to microvascular endothelial cells (19). Regardless of their origin, ECFC have attracted significant attention because of their potential for translational studies, gene therapy and vascular regeneration. In this review, we will discuss current applications and future perspectives of the use of ECFC in clinical translation (Figure 1).

## ECFC for the investigation of endothelial cell biology in disease

ECFC are an invaluable tool to study molecular endothelial dysfunction in disease, giving access to endothelial cells from patients and control groups in a non-invasive way. ECFC have been used for functional and molecular studies in disease (20). Differences in terms of ECFC phenotype and functional abilities have been attributed in many cases to genetic or epigenetic dysfunction. Moreover, the number of ECFC colonies can vary between different groups, although the significance of this parameter is still unclear. It is also unclear whether ECFC directly contribute to the pathogenesis of diseases. These studies open the opportunity for personalized approaches to characterize molecular and phenotypic defects in diseases with heterogenous clinical presentations. The main published literature is summarized below. Of note, interpretation and comparison across studies should be made with caution, as the ECFC isolation and culture methods vary between different laboratories, and this might affect the cells' proliferative capacity and possibly phenotype.

### Hematological disorders

#### Von willebrand disease (VWD)

ECFC have been used to study endothelial dysfunction in patients with von Willebrand Disease (VWD), the most common genetic bleeding disorder. VWD is caused by a defect or deficiency in von Willebrand factor (VWF), an endothelial and platelet protein essential for haemostasis. The disease is genetically heterogeneous, mostly due to mutations in the VWF gene; clinically, three main subtypes have been identified: type 1 and 3 with quantitative defects, and type 2 with qualitative defects (21). The classification can influence the therapeutic strategy, given that patients with type 1 VWD and cellular stores of VWF can be treated with an agent inducing VWF endothelial release (22), whilst patients with complete lack or dysfunction may require replacement therapy with plasma products. Using ECFC, it was possible for the first time to profile the synthesis and storage of VWF in endothelial cells from individual patients (23, 24), information which could be useful to influence the therapeutic choice. Besides its role in haemostasis, VWF has multiple vascular functions, including modulation of inflammation and angiogenesis (25). ECFC studies have confirmed that VWF plays a role in angiogenesis. In 2011, Starke et al. studied the *in vitro* angiogenic potential of ECFC isolated from type 1 and type 2 VWD patients and found overall significant enhancement of *in vitro* tube formation, proliferation and migration (26). Other studies have identified distinct defects in *in vitro* angiogenesis in ECFC from different VWD subtypes, and a significant degree of variability (27, 28). These studies highlight the great potential of ECFC to investigate processing and function of VWF in endothelial cells, and the need to standardize methods to be able to compare findings across laboratories.

#### Myeloproliferative neoplasms (MPN)

In patients with Philadelphia chromosome positive chronic myeloid leukemia (CML), a nine-fold increase in the number of ECFC colonies and increased proliferative potential was described compared to healthy donors (29). Analysis of *BCR/ABL1* revealed that all ECFC clones were *BCR/ABL1* negative and therefore were not clonally related to *BRC/ABL1*^+^ hematopoietic progenitors (29). Initial studies in patients with Philadelphia-negative myeloproliferative neoplasms identified the Janus kinase 2 V617F (JAK2^V617F^) mutation only in hematopoietic cells and not in ECFC (30). However, subsequent studies revealed a subgroup of patients with the JAK2^V617F^ mutation in ECFC, who had increased thrombotic events compared to patients without the mutation, suggesting that ECFC might be a useful tool to evaluate the thrombotic risk in these patients (31). Mechanism studies showed that the presence of the JAK2^V617F^ mutation in MPN ECFC was associated with hyper-phosphorylation of signal transducer and activator of transcription 3 (pSTAT-3) and STAT-5 (pSTAT-5), indicating abnormal activation of the JAK/STAT pathway in these cells. In addition, JAK2^V617F^-mutated ECFC showed increased adhesion to normal mononuclear cells (31). Further studies are required to determine whether JAK2^V617F^ mutated ECFC contribute to the increased risk of thrombosis in these patients or simply represent a biomarker of disease.

#### Sickle cell anemia

In children with sickle cell anemia, studies on ECFC were performed to investigate whether genetic differences affecting endothelial function could underlie the development of inflammatory vasculopathy causing stroke (occlusive disease at the circle of Willis) (32). Gene expression profiling of ECFC from children with vasculopathy demonstrated exaggerated responsiveness to inflammatory stimuli compared to ECFC from children without vasculopathy. Specifically, ECFC from the higher risk group showed increased RelA activation in response to stimulation with interleukin-1β/tumor necrosis factor α (TNF-α) (32). It would be interesting to determine whether these alterations are genetic or epigenetic, and whether this abnormal inflammatory response is ECFC-specific or a general response also affecting circulating immune cells.

#### Hereditary haemorrhagic telangiectasia (HHT)

Hereditary hemorrhagic telangiectasia (HHT) is caused by mutations in endoglin or activin receptor-like kinase-1 (ACVRL1/ALK1) genes and is an autosomal dominant vascular disorder. Isolation of ECFC from patients with HHT demonstrated reduced endoglin expression, impaired TGF-β signaling, disorganized cytoskeleton, and impaired angiogenic ability in a Matrigel assay compared to healthy controls (33). Further studies on ECFC from HHT patients revealed impaired ALK1- and ALK5-dependent TGF-β signaling, which promotes fragility of small vessels and vascular lesions; these may contribute to the clinical symptoms associated with this disease (34, 35).

#### Venous thromboembolic disease (VTD)

Two studies in patients with recurrent and unprovoked VTD have described dysfunctional ECFC; this might be linked to the increased risk of thrombotic events. The number of the ECFC colonies was increased in patients with VTD compared to control groups; moreover, electron microscopy studies revealed abnormalities of mitochondrial membrane, suggesting mitochondrial dysfunction (36). An increased pro-inflammatory cytokines profile was also found in the supernatants of cultures of ECFC from VTD patients (36). Further studies showed that ECFC from patients with VTD exhibit reduced proliferation, increased senescence, increased levels of reactive oxygen species (ROS) and impaired expression of ephrin-B2/Eph-4 genes, markers of venous and arterial endothelium involved in vascular regeneration (37). The abnormal inflammatory response, increased oxidative-stress and senescence detected in ECFC from patients with VTD might contribute to a defective response of the endothelium to vascular injury in these patients, possibly leading to recurrent thrombotic events. However, further studies are required to determine whether dysfunctional ECFC are involved in driving abnormal thrombus formation in these patients, or ECFC dysfunction in VTD is predominantly a biomarker.

### Pulmonary arterial hypertension (PAH)

Pulmonary arterial hypertension (PAH) is a condition characterized by the formation of plexiform lesions and concentric intimal fibrosis in small pulmonary arteries, with increased blood pressure in the arteries of the lungs. Hereditary PAH (HPAH) is often caused by mutations in the bone morphogenetic protein receptor type 2 gene (BMPRII). ECFC have been extensively used to investigate the pathogenesis of PAH. ECFC from PAH patients with BMPRII mutations demonstrated a hyperproliferative phenotype with impaired ability to form vascular networks (38), suggesting a pathogenetic role in the observed vascular remodeling. ECFC from patients with BMPRII mutations show a compromised signaling in response to the endothelial BMPRII ligand BMP9, which can be restored by agents such as chloroquine (39). BMP9 has been described as the preferred ligand for preventing apoptosis and enhancing monolayer integrity in ECFC from patients with BMPRII mutations (40), and has been used to reverse established PAH in preclinical models, suggesting new potential therapeutic approaches for inherited PAH. Proteomic screening in ECFC of HPAH patients with BMPRII mutations compared to healthy control subjects revealed translationally controlled tumor protein as a key mediator of endothelial prosurvival and growth signaling in PAH (41). MicroRNA and proteomic profiling of ECFC from patients with HPAH and idiopathic PAH demonstrated metabolic abnormalities, confirming a switch from oxidative phosphorylation to aerobic glycolysis (42). This is possibly due to down-regulation of miR-124 and its targets of Polypyrimidine Tract Binding Protein and Pyruvate Kinase M2 in PAH (42). Increased expression of chloride intracellular channel 4 (CLIC4) has been reported in ECFC from patients with idiopathic PAH, in line with evidence in pulmonary vessels (43). Increased CLIC4 expression activates nuclear factor-κB, followed by stabilization of hypoxia-inducible factor-1α and increased production of vascular endothelial growth factor (VEGF) and endothelin-1, contributing to endothelial dysfunction in PAH (43). Studies on ECFC have also supported the concept of a pathogenetic role for type I interferon in PAH. ECFC from PAH patients treated with interferon α show an abnormal increased release of interferon γ inducible protein 10 compared to healthy controls (44).

### Ischemic heart disease

ECFC studies in ischemic heart disease have multiple potential applications: as well as providing insights into the molecular mechanisms leading to endothelial dysfunction and how they can be targeted pharmacologically, ECFC are also being considered for autologous cell therapy for vascular regeneration (discussed later). The studies focusing on ECFC phenotype and/or function have so far yielded mixed findings, possibly because of the heterogeneity of this disease group. ECFC isolated from patients with stable ischemic heart failure demonstrated similar growth kinetics and neovascularization potential (*in vitro* and *in vivo*) compared to ECFC from control groups (young volunteers and age-matched controls) (45). In a model of hindlimb ischemia in nude mice, intramuscular injections of ECFC from patients with ischemic cardiomyopathy resulted in increased arteriogenesis similar to that observed with ECFC from healthy controls (45). Similar findings were confirmed in patients with premature coronary artery disease (46, 47). ECFC isolated from peripheral blood of patients with premature coronary disease showed no differences in the number of colonies or in the phenotype, function or microRNA expression profile, compared to healthy age and gender-matched controls (46). On the contrary, endothelial cells isolated from the vessel wall of the same patients exhibited impaired proliferation, adhesion and migration, and significantly reduced expression of microRNAs known to regulate endothelial function compared to controls (46), suggesting that in these patients circulating ECFC do not reflect the vascular dysfunction. Other studies focused on ECFC colony numbers. In patients referred for coronary angiography, higher numbers of ECFC colonies were associated with the presence of significant coronary artery disease, and ECFC number correlated with maximum angiographic stenosis severity (48). Interestingly, the onset of acute myocardial ischemia in a swine model resulted in rapid increase of ECFC numbers in the circulation compared to baseline levels, and mobilization of highly proliferative ECFC subpopulations (49). These findings suggest that ECFC could be acutely released in the circulation either from the damaged vascular tissue or in response to mobilizing factors secreted in the circulation due to myocardial ischemia (49). A subsequent study in patients with acute myocardial infarction (MI) vs. healthy controls confirmed increased ECFC numbers in the circulation within 3 h from the onset of symptoms (50). More recently, successful isolation of ECFC colonies in patients within 12 h from the onset of acute MI (51) was correlated with outcome. The presence of ECFC colonies was associated with reduced microvascular obstruction, infarct size and left ventricular remodeling, suggesting that circulating ECFC may be a marker of preserved microvascular integrity in patients with acute MI (51). In these two studies, molecular and functional analysis of ECFC was not performed. Future studies on targeted patient populations will be required to understand the significance and possible role of ECFC in ischemic heart disease.

### Chronic lung disease in adults

#### Chronic obstructive pulmonary disease (COPD)

COPD is a chronic inflammatory lung disease that results in largely irreversible and progressive airflow limitation. It is caused by long-term exposure to irritating gases, most often from cigarette smoke. We isolated ECFC from smokers and patients with COPD to study endothelial dysfunction in these groups that have increased risk of cardiovascular disease. ECFC from smokers and COPD patients exhibited accelerated aging due to epigenetic dysfunction (52). This involved reduction of sirtuin-1 (SIRT1), a protein deacetylase that protects against DNA damage, due to activation of ataxia telangiectasia-mutated (ATM) kinase, which plays a key role in DNA damage response. Impaired angiogenic ability of ECFC from COPD patients was shown in an *in vivo* Matrigel assay in nude mice. Importantly, targeted pharmacologically treatment was able to reduce the increased senescence in ECFC from COPD patients (52). Further studies demonstrated microRNA dysregulation in ECFC from COPD, affecting the miR-126-3p, a critical microRNA in vascular development, endothelial homeostasis and inflammation (53). We found that miR-126-3p is downregulated in ECFC from smokers and COPD patients and promotes an augmented DNA damage response through activation of ATM, contributing to endothelial senescence and dysfunction in these groups (54).

#### Idiopathic pulmonary fibrosis (IPF)

IPF is a rare lung disease characterized by progressive scarring of the lungs and is associated with pulmonary vascular remodeling. ECFC isolated from patients with IPF compared to a control group showed no difference in terms of number of colonies or proliferative potential (55). However, subgroup analysis of the IPF patients showed increased number of ECFC colonies in IPF patients with significantly impaired gas transfer [Diffusing capacity of the lung for the carbon monoxide (DLco) <40%]. Also, ECFC proliferation was increased in patients with exacerbation compared to stable disease (55). Another study suggested that the vascular remodeling in fibrotic lung diseases might be regulated by cooperation of ECFC with fibrocytes, cells that coexpress hematopoietic and fibroblast markers and contribute to organ fibrosis (56). Further studies demonstrated increased microparticles released from ECFC isolated from IPF patients compared to controls; these exhibited increased plasminogen activation and could stimulate fibroblast migration (57), suggesting a pathogenetic role of ECFC-derived endothelial microparticles to pulmonary fibrogenesis.

### Diabetes

Most studies of ECFC in diabetes have investigated cord blood ECFC from gestational diabetes mellitus (GDM) pregnancies. The first study published by Ingram et al. demonstrated that cord blood ECFC from diabetic pregnancies exhibited premature senescence and impaired proliferation *in vitro*, and reduced vasculogenic potential *in vivo* compared to cord blood ECFC from uncomplicated pregnancies (58). Microarray screen and validation of selected genes identified epigenetic dysfunction, involving DNA methylation of placenta-specific 8 as contributor to increased senescence and reduced proliferation of ECFC from GDM compared to control pregnancies (59). ECFC isolated from peripheral blood from patients with type 2 diabetes demonstrated reduced *in vitro* proliferation rate and importantly reduced neovascularization capacity *in vivo* compared to nondiabetic ECFC (60). Interestingly, pre-treatment of diabetic ECFC with globular adiponectin was able to improve neovascularization in a murine hind limb ischemia model (60).

### Premature neonates, pregnancy-related disease, and associated comorbidities

It is well established that ECFC from full-term cord blood emerge earlier in culture, proliferate faster and show enhanced vasculogenic ability compared to ECFC from adult peripheral blood (9, 61, 62). In contrast, numerous studies describe reduced numbers and dysfunction of ECFC isolated from low birth weight (LBW) or preterm (PT) infants, linked to associated comorbidities such as bronchopulmonary dysplasia (BPD) and increased risk of cardiovascular diseases in adulthood. The yield of ECFC colonies has been found reduced in PT infants (24–28 weeks) compared with term controls, whereas gestational age of 33–36 weeks yielded ECFC colonies at equivalent numbers to term infants (63). The angiogenic properties of ECFC have been found impaired in LBW preterm neonates *in vitro* and *in vivo* (64). Transcriptome profiling of LBW ECFC demonstrated an increased expression of antiangiogenic genes including thrombospondin 1) (64). The same group subsequently showed that ECFC isolated from PT infants display accelerated senescence due to reduced expression of SIRT1, and that SIRT1 overexpression could restore PT.

ECFC angiogenic ability *in vivo* (65). SIRT1 deficiency in PT ECFC was also found to regulate the biogenesis of microparticles, mediating a paracrine induction of senescence in naïve endothelial cells (66). These findings suggest a novel link between epigenetic dysfunction leading to ECFC senescence and increased cardiovascular risk in individuals born preterm.

Maternal characteristics and pregnancy related disease might also affect ECFC isolated from cord blood. For example, a positive correlation between pre-pregnancy maternal body mass index and ECFC yield has been described, which was independent of other obstetric factors (67). In preeclampsia, the number of cord blood ECFC was reduced compared to control pregnancies; however, ECFC function was similar between preeclampsia patients and controls, including the ability to form vascular networks *in vivo* (68). As mentioned earlier, gestational diabetes can also affect ECFC levels and function (58, 59).

#### Bronchopulmonary dysplasia (BPD)

BPD is a chronic lung disease mainly affecting low birth weight premature infants treated with supplemental oxygen and mechanical ventilation for respiratory distress syndrome. It associates with impaired vascular and alveolar growth. Several studies suggest a pathogenetic role of ECFC in preterm infants. Increased number of ECFC colonies have been found in preterm cord blood; these cells exhibit increased growth but also increased apoptotic susceptibility to hyperoxia compared to ECFC from term cord blood (69). This effect might be caused to disruption of VEGF-nitric oxide signaling (70). Additional studies have demonstrated that the number of cord blood ECFC in preterm babies who developed moderate or severe BPD were significantly reduced (71, 72), suggesting that a low number of ECFC may contribute to abnormal vascular repair in preterm infants resulting in BPD.

## ECFC for autologous cell therapy for vascular repair

ECFC represent the most potent vascular reparative cell type among all putative “EPC,” the only cell population with clonal proliferative potential and ability to form blood vessels *in vivo* (9, 10, 73). Numerous preclinical *in vivo* studies have demonstrated the vascular reparative effect of ECFC in disease models (74). Several mechanism of action have been suggested: (a) direct incorporation of ECFC at sites of vascular damage and formation of blood vessels (61, 75), (b) paracrine effect, mediated by the release of pro-angiogenic growth factors or extracellular vesicles secreted from ECFC (76–79), (c) support of the reparative ability of other cells, such as mesenchymal progenitor cells (MPC) (80–82) or adipose stromal cells (ASC) (83). A combined mechanism of action has been suggested in several studies, showing direct incorporation of ECFC in the blood vessels and paracrine effect on other reparative cells (84, 85). However, in many studies the exact mechanism of action remains elusive. Below we review the evidence supporting the use of ECFC for autologous cell therapy and comment on the proposed mechanism of action.

ECFC have been used as cell therapy in several preclinical models of ischemic vascular diseases. They have been shown to promote neovascularization in numerous *in vivo* models of hind limb ischemia (84, 86–90) and MI (91). Direct incorporation of ECFC in the ischemic tissue has been demonstrated (84, 87, 88), and shown to be of significant importance for the functional recovery of the ischemic tissue (86). The co-implantation of ECFC with MPC further improved neovascularization of ischemic tissue compared to ECFC alone (84). However, whether this is due to direct involvement of ECFC and MPC in new blood vessels formation, or to ECFC and MPC-derived factors stimulating endogenous neovascularization is not clear (84). The use of ECFC in models of ischemic stroke (85, 92) and traumatic brain injury (93, 94) suggests that ECFC might be a promising treatment to improve tissue viability and function by promoting angiogenesis and neurogenesis. ECFC homing has been confirmed at sites of ischemic brain tissue (85, 92, 93). However, engraftment of ECFC into neovessels was limited and a therapeutic effect via a paracrine mechanism has been suggested (85).

ECFC cell therapy may also promote vascular repair and revascularization of ischemic retinopathies, such as diabetic retinopathy and retinopathy of prematurity. Medina et al. using a murine model of retinal ischemia, demonstrated that ECFC could incorporate into the retinal microvascular tube network and promote vascular repair (95). ECFC might also exert a vasoregenerative effect in ischemic retinopathies via a paracrine way, mediated at least in part by extracellular vesicles containing microRNA that promote angiogenesis (96). Optimization studies of ECFC for cell therapy have identified a low dose of ECFC which was effective both by local intravitreal injection and systemic intracarotid delivery, without any significant toxicity (97).

ECFC autologous cell therapy might also exert vascular reparative effects in preclinical models of PAH and BPD (98). In a model of hyperoxia-induced BPD, intravenous administration of cord blood ECFC was able to reverse the alveolar growth arrest, preserved lung vascularity, and attenuated PAH (98). The observation of low ECFC engraftment and the protective effect of ECFC-derived conditioned media suggest a paracrine mechanism of action (98); this is also supported by other studies in experimental models of BPD showing prevention of PAH by ECFC-derived conditioned media (76).

ECFC cell therapy might also exert a therapeutic effect in acute kidney injury (AKI). Preclinical models have shown that use of ECFC can mitigate the severity of disease and preserve vascular function (77, 78, 99, 100). In most studies the effect is mediated by secreted factors from ECFC and not cell engraftment. In mice with ischemic AKI, intravenous administration of ECFC significantly attenuated increases in plasma creatinine, tubular necrosis, macrophage infiltration, oxidative stress and apoptosis, without cell engraftment in the kidneys (77). Administration of ECFC conditioned media or ECFC-derived exosomes also exerted a protective effect, indicating that the effects of cord blood ECFC are due to ECFC-derived exosomes (77, 78). Further studies demonstrated that ECFC exosomes were enriched in miR-486-5p, which targets the phosphatase and tensin homolog (PTEN) (79). In mice with ischemic kidney disease, infusion of ECFC exosomes reduced kidney injury via transfer of miR-486-5p targeting PTEN (79).

## ECFC for gene therapy

ECFC have being used as cellular vehicle for gene therapy. Important advantages of ECFC compared to other cell types include non-invasive isolation and expansion from peripheral blood, genomic stability in cell culture and ease of genetic manipulation. Preclinical studies that have used ECFC as vehicles for gene therapy are the following.

### Hemophilia A

Autologous ECFC have been successfully used for gene therapy in animal models of hemophilia A (101, 102). ECFC isolated from canine blood were transduced with a lentiviral vector encoding for the canine B-domain deleted Factor VIII transgene and were implanted subcutaneously in a Matrigel scaffold or into the omentum. Therapeutic levels of Factor VIII persisted over a long period of time, more than 15 weeks in Matrigel scaffolds and up to a year with omentum implantation, indicating ECFC as promising vehicle for gene therapy in hemophilia A (101, 103).

### VWD

Gene therapy using ECFC has been considered for treatment of VWD. Development of gene therapy for VWD has been hampered by the considerable length of the VWF cDNA and posttranslational processing of the protein. ECFC isolated from type 3 VWD dogs were transduced with a lentiviral vector encoding complete human VWF; this resulted in high-transduction efficiencies and expression of functional vector-encoded VWF (104).

### Anemia

In patients with advanced-stage chronic kidney disease, treatment of chronic anemia requires routine injections of recombinant erythropoietin (EPO). Using genetically engineered ECFC, Melero-Martin's group managed to express EPO under the control of a tetracycline-regulated system and generated subcutaneous vascular networks capable of systemic EPO release in immunodeficient mice bloodstream (105). After activation of EPO expression, erythropoiesis was induced in mice, a process that was completely reversible, indicating delivery of EPO by genetically modified ECFC as a potential therapeutic approach in patients requiring frequent EPO injections.

### Cancer

ECFC have been also considered as carriers for systemic cancer gene therapy. The principle is to use genetically engineered ECFC to deliver angiogenic inhibitors or other suicide genes directly into the tumor endothelium (106–108). Systemic delivery of ECFC expressing fms-like tyrosine kinase-1 and/or angiostatin-endostatin fusion protein was able to inhibit tumor growth, reduced tumor volume and increased survival in mice cancer models (106, 107), indicating ECFC as potential candidates for tumor-specific delivery of cancer gene therapy.

## ECFC for tissue bioengineering

Development of vascular networks is an essential goal for regenerative medicine and may be a future approach for the treatment of ischemic vascular disease. ECFC are currently being explored for tissue engineering strategies for tissue repair or regeneration.

### Bioartificial vascular structures

Artificial vascularization of engineered biocompatible scaffolds is essential, since a major challenge in tissue engineering is to supply bioengineered tissue transplants with sufficient amounts of nutrients and oxygen and to allow metabolite removal. ECFC is an attractive approach for engineering bioartificial vascular structures, due to their proven advanced vasculogenetic abilities (74, 81, 100). However, scaffold materials might have a significant effect on ECFC's functional abilities. For example, Critser et al. showed that the physical properties of collagen matrices influence ECFC-dependent vasculogenesis *in vivo* (109). There are several natural and biocompatible synthetic matrices able to support the development of vascular networks and tissue regeneration with ECFC (110–117). These matrices which mimic the natural extracellular matrix have been proven safe for clinical use, and can be modulated by changing the biochemical and physiological properties of the scaffold or by using growth factors such as VEGF, Angiopoietin 1, or BMP-2, promoting the pro-angiogenic abilities of the cells (74).

### Wound healing

Vascularization is critical for wound healing, a process that involves the interaction of multiple cells, including platelets, fibroblasts, keratinocytes, and endothelial cells. ECFC have been used to support vascularization during wound healing using different scaffolds and tissue-engineered human skin substitutes (118–120). ECFC seeded in appropriate scaffold were able to promote wound healing when compared with intradermal ECFC injection (118). Use of ECFC in tissue-engineered human skin substitutes promoted the formation of vascular conduits enabling perfusion and survival of human bioengineered tissues (119). Furthermore, integration of ECFC in dermal fibroblast sheets induced robust vasculogenesis, accelerated epithelial coverage and matrix organization of the wound bed and prevented wound contraction (120). The therapeutic effects were due to both active incorporation of ECFC into new vessels and to trophic stimulation of host angiogenesis by growth factors secreted by ECFC, such as placental growth factor (120).

### Bone repair

ECFC have been also investigated for bone repair. Vascularization is crucial for successful bone regeneration and for successful use of tissue engineered bone constructs, as a rapid connection to the vascular network is required. Subcutaneous implantation of cord blood ECFC with human fetal MSC induced formation of chimeric human vessels and ectopic bone formation in immunodeficient mice after 12 weeks (113). Cord blood ECFC with biphasic calcium phosphate/BMP-2 bone tissue engineering constructs demonstrated *in vivo* vasculogenesis within the macropore space of the constructs, suggesting that scaffolds containing ECFC have significant potential for advanced neovascularization in bone defects (114). Subcutaneous application of co-culture of ECFC with human primary osteoblasts on starch polycaprolactone fiber meshes induced formation of ECFC-derived blood vessels integrated into the host tissue and anastomosed to the vascular supply (116). These studies suggest that ECFC might have an important role for bone regeneration.

## Improving ECFC therapeutic efficiency

As mentioned earlier, ECFC from patients with different diseases often present a dysfunctional phenotype with low proliferative potential, increased cellular senescence, a proinflammatory profile and reduced vasculogenic abilities, in many cases attributed to genetic or epigenetic dysfunction. Some of the key studies on how to improve ECFC therapeutic efficacy in view of their use for autologous cell therapy are summarized below.

### Epigenetic activation

Recent studies have investigated the epigenetic profile of cord blood ECFC and found that key proangiogenic pathways are repressed, reducing ECFC's regenerative potential (121, 122). Importantly, these studies show that epigenetic drugs including HDAC inhibitors can lift this repression and activate pro-angiogenic signaling pathways, improving ECFC vasculogenic ability *in vivo* (89, 121, 122). Epigenetic dysfunction involving the histone deacetylase SIRT1 has been also demonstrated in disease, for example in COPD or ECFC from preterm infants, contributing to ECFC senescence and impaired angiogenic ability of them (52, 65). Targeting epigenetic dysfunction with *ex vivo* pre-treatment could partially restore ECFC senescence and dysfunction (52, 65), suggesting that *ex vivo* epigenetic activation of ECFC might improve their therapeutic efficacy if needed for autologous cell regenerative purposes.

### ECFC pre-treatment with bioactive compounds/agents

Numerous studies have demonstrated that pre-treatment of ECFC can improve their therapeutic efficacy. For example, pretreatment of dysfunctional ECFC from type 2 diabetes patients with globular adiponectin enhanced neovascularization in murine models of hindlimb ischemia, in both normoglycemic and hyperglycemic conditions (60). Priming of ECFC with EPO before intravenous injection increased revascularization in a hindlimb ischemia mouse model (123). Similarly, fucoidan-pretreatment was found to enhance survival, proliferation, incorporation and endothelial differentiation of senescent ECFC transplanted in a murine hind limb ischemia model (124). Transplantation of genistein-pretreated ECFC into myocardial ischemic sites enhanced neovascularization, decreased myocardial fibrosis and improved cardiac function (91). Implantation of ECFC with human platelet lysate promoted vasculogenesis and augmented blood vessel formation via diminishing apoptosis of the implanted ECFC *in vivo* (125). Transmembrane TNF-α expression is increased on the surface of highly proliferative ECFC, regulating their proliferative capacity, and might be another candidate pathway for enhancing ECFC angiogenic ability (126). For an extended list of compounds/agents having an effect on ECFC functional abilities please see reference from Tasev et al. (74).

## Challenges that limit ECFC clinical applications

Current challenges that need to be addressed for the use of ECFC in clinical trials are summarized below.

### Time and efficacy of isolation and expansion of ECFC from peripheral blood

ECFC cannot be successfully isolated from peripheral blood from all individuals. This is likely due to low number of circulating ECFC present in the blood, and this can be further reduced in several diseases, as described above. Currently there are no markers that uniquely identify human ECFC, which could be used to enrich for these cells (62, 100). The recent identification of tissue-resident vascular endothelial stem cells, positive for CD157, that can be clonally expanded and contribute to angiogenesis and maintenance of blood vessels *in vivo*, is a promising achievement toward this direction (127).

### Replacement of reagents derived from animals

Most protocols for ECFC isolation utilize reagents derived from animals, such as fetal bovine serum. Further optimization of protocols that use human platelet lysates (125, 128, 129) or autologous human serum (130) may provide the required alternative to generate ECFC compatible with good manufacturing practice (GMP) standards in cellular therapy facilities.

### Limited availability of ECFC

Regenerative approaches usually require large numbers of cells, which in many cases is difficult to obtain from peripheral blood ECFC. Generation of iPSC-derived ECFC (14) could be used to obtain large amounts of autologous ECFC-like cells for vascular regeneration. However, the process of generating patient specific iPSC-derived ECFC is complex and time-consuming (14, 100). Moreover, no data is available yet on whether these cells recapitulate the functional and transcriptional profile of “true” ECFC and whether the “ECFC” phenotype is stable. Isolation of ECFC from white adipose tissue vasculature (131) might provide another practical alternative to obtain large amounts of autologous ECFC for vascular regeneration.

## Conclusions

It is evident from the current literature that significant progress has been achieved from the early days of “EPC” ambiguity, a term which included various cell populations of different origin. ECFC have been widely recognized as an important tool to study endothelial molecular dysfunction in disease and a promising tool for vascular regenerative approaches and gene therapy. There is plenty of evidence of the importance of these cells for multiple applications. Future studies will address current obstacles and help develop the use ECFC in clinical settings for regenerative approaches.

## Author contributions

KP: conception and design, manuscript writing. AR: conception and design, financial support, contributed to manuscript writing.

### Conflict of interest statement

The authors declare that the research was conducted in the absence of any commercial or financial relationships that could be construed as a potential conflict of interest.

## References

[1] Asahara T Murohara T Sullivan A Silver M van der Zee R Li T. Isolation of putative progenitor endothelial cells for angiogenesis. Science (1997) 275:964–7. 10.1126/science.275.5302.964 9020076

[2] Lin Y Weisdorf DJ Solovey A Hebbel RP. Origins of circulating endothelial cells and endothelial outgrowth from blood. J Clin Invest. (2000) 105:71–7. 10.1172/JCI8071 10619863 PMC382587

[3] Yoder MC. Human endothelial progenitor cells. Cold Spring Harb Perspect Med. (2012) 2:a006692. 10.1101/cshperspect.a006692 22762017 PMC3385946

[4] Medina RJ Barber CL Sabatier F Dignat-George F Melero-Martin JM. Endothelial progenitors: a consensus statement on nomenclature. Stem Cells Transl Med. (2017) 6:1316–20. 10.1002/sctm.16-0360 28296182 PMC5442722

[5] Mund JA Estes ML Yoder MC Ingram DA Jr Case J. Flow cytometric identification and functional characterization of immature and mature circulating endothelial cells. Arterioscler Thrombo Vasc Biol. (2012) 32:1045–53. 10.1161/ATVBAHA.111.244210 22282356 PMC3306529

[6] Estes ML Mund JA Ingram DA Case J. Identification of endothelial cells and progenitor cell subsets in human peripheral blood. Curr Protoc Cytom. (2010) 9:Unit 9.33.1–11. 10.1002/0471142956.cy0933s52 20373498

[7] Asahara T Kawamoto A Masuda H. Concise review: circulating endothelial progenitor cells for vascular medicine. Stem Cells (Dayton, Ohio) (2011) 29:1650–5. 10.1002/stem.745 21948649

[8] Medina RJ O'Neill CL O'Doherty TM Knott H Guduric-Fuchs J. Myeloid angiogenic cells act as alternative M2 macrophages and modulate angiogenesis through interleukin-8. Mol Med. (2011) 17:1045–55. 10.2119/molmed.2011.00129 21670847 PMC3188859

[9] Ingram DA Mead LE Tanaka H Meade V Fenoglio A Mortell K. Identification of a novel hierarchy of endothelial progenitor cells using human peripheral and umbilical cord blood. Blood (2004) 104:2752–60. 10.1182/blood-2004-04-1396 15226175

[10] Ingram DA Mead LE Moore DB Woodard W Fenoglio A Yoder MC. Vessel wall-derived endothelial cells rapidly proliferate because they contain a complete hierarchy of endothelial progenitor cells. Blood (2005) 105:2783–6. 10.1182/blood-2004-08-3057 15585655

[11] Duong HT Comhair SA Aldred MA Mavrakis L Savasky BM Erzurum SC Asosingh K. Pulmonary artery endothelium resident endothelial colony-forming cells in pulmonary arterial hypertension. Pulmonary Circ. (2011) 1:475–86. 10.4103/2045-8932.93547 22530103 PMC3329078

[12] Alphonse RS Vadivel A Zhong S McConaghy S Ohls R Yoder MC. The isolation and culture of endothelial colony-forming cells from human and rat lungs. Natu Protoc. (2015) 10:1697–708. 10.1038/nprot.2015.107 26448359

[13] Solomon O'Reilly M Ionescu L Alphonse RS Rajabali S Zhong S. Functional differences between placental micro- and macrovascular endothelial colony-forming cells. Stem Cells Transl Med. (2016) 5:291–300. 10.5966/sctm.2014-0162 26819255 PMC4807658

[14] Prasain N Lee MR Vemula S Meador JL Yoshimoto M Ferkowicz MJ. Differentiation of human pluripotent stem cells to cells similar to cord-blood endothelial colony-forming cells. Nat Biotechnol. (2014) 32:1151–7. 10.1038/nbt.3048 25306246 PMC4318247

[15] Tura O Skinner EM Barclay GR Samuel K Gallagher RC Brittan M. Late outgrowth endothelial cells resemble mature endothelial cells and are not derived from bone marrow. Stem Cells (Dayton, Ohio) (2013) 31:338–48. 10.1002/stem.1280 23165527

[16] Mondor Jorquera A Sene C Adriouch S Adams RH Zhou B. Clonal proliferation and stochastic pruning orchestrate lymph node vasculature remodeling. Immunity (2016) 45:877–88. 10.1016/j.immuni.2016.09.017 27760341

[17] Manavski Y Lucas T Glaser SF Dorsheimer L Gunther S Braun T. Clonal expansion of endothelial cells contributes to ischemia-induced neovascularization. Circ Res. (2018) 122:670–7. 10.1161/CIRCRESAHA.117.312310 29358229

[18] Corey DM Rinkevich Y Weissman IL. Dynamic patterns of clonal evolution in tumor vasculature underlie alterations in lymphocyte-endothelial recognition to foster tumor immune escape. Cancer Res. (2016) 76:1348–53. 10.1158/0008-5472.CAN-15-1150 26719541 PMC4794394

[19] Toshner M Dunmore BJ McKinney EF Southwood M Caruso P Upton PD. Transcript analysis reveals a specific HOX signature associated with positional identity of human endothelial cells. PLoS ONE (2014) 9:e91334. 10.1371/journal.pone.0091334 24651450 PMC3961275

[20] Medina RJ O'Neill CL O'Doherty TM Wilson SE Stitt AW. Endothelial progenitors as tools to study vascular disease. Stem Cells Int. (2012) 2012:346735. 10.1155/2012/346735 22550504 PMC3329655

[21] Lillicrap D. von Willebrand disease: advances in pathogenetic understanding, diagnosis, and therapy. Blood (2013)122:3735–40. 10.1182/blood-2013-06-498303 24065240 PMC3952678

[22] Mannucci PM. Treatment of von Willebrand disease. Thrombo Haemost. (2001)86:149–53. 10.1055/s-0037-1616212 11487002

[23] Starke RD Paschalaki KE Dyer CE Harrison-Lavoie KJ Cutler JA. Cellular and molecular basis of von Willebrand disease: studies on blood outgrowth endothelial cells. Blood (2013) 121:2773–84. 10.1182/blood-2012-06-435727 23355534 PMC3617637

[24] Wang JW Bouwens EA Pintao MC Voorberg J Safdar H Valentijn KM. Analysis of the storage and secretion of von Willebrand factor in blood outgrowth endothelial cells derived from patients with von Willebrand disease. Blood (2013) 121:2762–72. 10.1182/blood-2012-06-434373 23426949

[25] Lenting PJ Casari C Christophe OD Denis CV. von Willebrand factor: the old, the new and the unknown. J Thrombo Haemost. (2012) 10:2428–37. 10.1111/jth.12008 23020315

[26] Starke RD Ferraro F Paschalaki KE Dryden NH McKinnon TA. Endothelial von Willebrand factor regulates angiogenesis. Blood (2011) 117:1071–80. 10.1182/blood-2010-01-264507 21048155 PMC3035068

[27] Groeneveld DJ van Bekkum T Dirven RJ Wang JW Voorberg J Reitsma PH. Angiogenic characteristics of blood outgrowth endothelial cells from patients with von Willebrand disease. J Thrombo Haemost. (2015) 13:1854–66. 10.1111/jth.13112 26270243

[28] Selvam SN Casey LJ Bowman ML Hawke LG Longmore AJ Mewburn J. Abnormal angiogenesis in blood outgrowth endothelial cells derived from von Willebrand disease patients. Blood Coagul Fibrinolys. (2017) 28:521–33. 10.1097/MBC.0000000000000635 28362648 PMC5960581

[29] Otten J Schultze A Schafhausen P Otterstetter S Dierlamm J Bokemeyer C. Blood outgrowth endothelial cells from chronic myeloid leukaemia patients are BCR/ABL1 negative. Br J Haematol. (2008) 142:115–8. 10.1111/j.1365-2141.2008.07195.x 18477035

[30] Piaggio G Rosti V Corselli M Bertolotti F Bergamaschi G Pozzi S. Endothelial colony-forming cells from patients with chronic myeloproliferative disorders lack the disease-specific molecular clonality marker. Blood (2009) 114:3127–30. 10.1182/blood-2008-12-190991 19628707

[31] Teofili L Martini M Iachininoto MG Capodimonti S Nuzzolo ER Torti L. Endothelial progenitor cells are clonal and exhibit the JAK2(V617F) mutation in a subset of thrombotic patients with Ph-negative myeloproliferative neoplasms. Blood (2011) 117:2700–7. 10.1182/blood-2010-07-297598 21212285

[32] Chang Milbauer L Wei P Enenstein J Jiang A Hillery CA Scott JP. Genetic endothelial systems biology of sickle stroke risk. Blood (2008) 111:3872–9. 10.1182/blood-2007-06-097188 18156497 PMC2275038

[33] Fernandez LA Sanz-Rodriguez F Zarrabeitia R Perez-Molino A Hebbel RP. Blood outgrowth endothelial cells from hereditary haemorrhagic telangiectasia patients reveal abnormalities compatible with vascular lesions. Cardiovasc Res. (2005) 68:235–48. 10.1016/j.cardiores.2005.06.009 15993872

[34] Fernandez LA Sanz-Rodriguez F Zarrabeitia R Perez-Molino A Morales C Restrepo CM. Mutation study of Spanish patients with hereditary hemorrhagic telangiectasia and expression analysis of Endoglin and ALK1. Hum Mutat. (2006) 27:295. 10.1002/humu.9413 16470589

[35] Fernandez LA Sanz-Rodriguez F Blanco FJ Bernabeu C Botella LM Hereditary hemorrhagic telangiectasia, a vascular dysplasia affecting the TGF-beta signaling pathway. Clin Med Res. (2006) 4:66–78. 10.3121/cmr.4.1.66 16595794 PMC1435660

[36] Alvarado-Moreno JA Hernandez-Lopez R Chavez-Gonzalez A Yoder MC Rangel-Corona R Isordia-Salas I Endothelial colony-forming cells: biological and functional abnormalities in patients with recurrent, unprovoked venous thromboembolic disease. Thrombosis Res. (2016) 137:157–68. 10.1016/j.thromres.2015.11.005 26597044

[37] Hernandez-Lopez R Chavez-Gonzalez A Torres-Barrera P Moreno-Lorenzana D Lopez-DiazGuerrero N Santiago-German D. Reduced proliferation of endothelial colony-forming cells in unprovoked venous thromboembolic disease as a consequence of endothelial dysfunction. PLoS ONE (2017) 12:e0183827. 10.1371/journal.pone.0183827 28910333 PMC5598948

[38] Toshner M Voswinckel R Southwood M Al-Lamki R Howard LS Marchesan D. Evidence of dysfunction of endothelial progenitors in pulmonary arterial hypertension. Am J Respir Critic Care Med. (2009) 180:780–7. 10.1164/rccm.200810-1662OC 19628780 PMC2778151

[39] Dunmore BJ Drake KM Upton PD Toshner MR Aldred MA Morrell NW. The lysosomal inhibitor, chloroquine, increases cell surface BMPR-II levels and restores BMP9 signalling in endothelial cells harbouring BMPR-II mutations. Human Mol Genet. (2013) 22:3667–79. 10.1093/hmg/ddt216 23669347 PMC3749859

[40] Long L Ormiston ML Yang X Southwood M Graf S Machado RD. Selective enhancement of endothelial BMPR-II with BMP9 reverses pulmonary arterial hypertension. Nat Med. (2015) 21:777–85. 10.1038/nm.3877 26076038 PMC4496295

[41] Lavoie JR Ormiston ML Perez-Iratxeta C Courtman DW Jiang B Ferrer E. Proteomic analysis implicates translationally controlled tumor protein as a novel mediator of occlusive vascular remodeling in pulmonary arterial hypertension. Circulation (2014) 129:2125–35. 10.1161/CIRCULATIONAHA.114.008777 24657995

[42] Caruso P Dunmore BJ Schlosser K Schoors S Dos Santos C Perez-Iratxeta C. Identification of microRNA-124 as a major regulator of enhanced endothelial cell glycolysis in pulmonary arterial hypertension via PTBP1 (Polypyrimidine Tract Binding Protein) and Pyruvate Kinase M2. Circulation (2017) 136:2451–67. 10.1161/CIRCULATIONAHA.117.028034 28971999 PMC5736425

[43] Wojciak-Stothard B Abdul-Salam VB Lao KH Tsang H Irwin DC Lisk C. Aberrant chloride intracellular channel 4 expression contributes to endothelial dysfunction in pulmonary arterial hypertension. Circulation (2014) 129:1770–80. 10.1161/CIRCULATIONAHA.113.006797 24503951 PMC4033409

[44] George PM Oliver E Dorfmuller P Dubois OD Reed DM Kirkby NS. Evidence for the involvement of type I interferon in pulmonary arterial hypertension. Circ Res. (2014) 114:677–88. 10.1161/CIRCRESAHA.114.302221 24334027 PMC4006084

[45] Dauwe D Pelacho B Wibowo A Walravens AS Verdonck K Gillijns H. Neovascularization potential of blood outgrowth endothelial cells from patients with stable ischemic heart failure is preserved. J Am Heart Assoc. (2016) 5:e002288. 10.1161/JAHA.115.002288 27091182 PMC4843533

[46] Brittan M Hunter A Boulberdaa M Fujisawa T Skinner EM Shah AS. Impaired vascular function and repair in patients with premature coronary artery disease. Eur J Prevent Cardiol. (2015) 22:1557–66. 10.1177/2047487315600169 26276790

[47] Stroncek JD Grant BS Brown MA Povsic TJ Truskey GA Reichert WM. Comparison of endothelial cell phenotypic markers of late-outgrowth endothelial progenitor cells isolated from patients with coronary artery disease and healthy volunteers. Tissue Eng Part A (2009) 15:3473–86. 10.1089/ten.tea.2008.0673 19435420 PMC2792057

[48] Guven H Shepherd RM Bach RG Capoccia BJ Link DC. The number of endothelial progenitor cell colonies in the blood is increased in patients with angiographically significant coronary artery disease. J Am Coll Cardiol. (2006) 48:1579–87. 10.1016/j.jacc.2006.04.101 17045891

[49] Huang L Hou D Thompson MA Baysden SE Shelley WC Ingram DA. Acute myocardial infarction in swine rapidly and selectively releases highly proliferative endothelial colony forming cells (ECFCs) into circulation. Cell Transpl. (2007) 16:887–97. 10.3727/096368907783338181 18293887

[50] Massa M Campanelli R Bonetti E Ferrario M Marinoni B Rosti V. Rapid and large increase of the frequency of circulating endothelial colony-forming cells (ECFCs) generating late outgrowth endothelial cells in patients with acute myocardial infarction. Exp Hematol. (2009) 37:8–9. 10.1016/j.exphem.2008.09.007 19013004

[51] Meneveau N Deschaseaux F Seronde MF Chopard R Schiele F Jehl J. Presence of endothelial colony-forming cells is associated with reduced microvascular obstruction limiting infarct size and left ventricular remodelling in patients with acute myocardial infarction. Basic Res Cardiol. (2011) 106:1397–410. 10.1007/s00395-011-0220-x 21904841

[52] Paschalaki KE Starke RD Hu Y Mercado N Margariti A Gorgoulis VG. Dysfunction of endothelial progenitor cells from smokers and chronic obstructive pulmonary disease patients due to increased DNA damage and senescence. Stem Cells (Dayton, Ohio) (2013)31:2813–26. 10.1002/stem.1488 23897750 PMC4377082

[53] Fish JE Santoro MM Morton SU Yu S Yeh RF Wythe JD. miR-126 regulates angiogenic signaling and vascular integrity. Dev Cell (2008) 15:272–84. 10.1016/j.devcel.2008.07.008 18694566 PMC2604134

[54] Paschalaki KE Zampetaki A Baker JR Birrell MA Starke RD Belvisi MG. Downregulation of microRNA-126 augments DNA damage response in cigarette smokers and COPD patients. Am J Respir Crit Care Med. (2018). 197:665–8. 10.1164/rccm.201706-1304LE 28753388 PMC6005241

[55] Smadja DM Mauge L Nunes H d'Audigier C Juvin K Borie R. Imbalance of circulating endothelial cells and progenitors in idiopathic pulmonary fibrosis. Angiogenesis (2013) 16:147–57. 10.1007/s10456-012-9306-9 22983452

[56] Smadja DM Dorfmuller P Guerin CL Bieche I Badoual C Boscolo E. Cooperation between human fibrocytes and endothelial colony-forming cells increases angiogenesis via the CXCR4 pathway. Thrombo Haemost. (2014) 112:1002–13. 10.1160/th13-08-0711 25103869 PMC4751883

[57] Bacha NC Blandinieres A Rossi E Gendron N Nevo N Lecourt S. Endothelial microparticles are associated to pathogenesis of idiopathic pulmonary fibrosis. Stem Cell Rev. (2018) 14:223–35. 10.1007/s12015-017-9778-5 29101610

[58] Ingram DA Lien IZ Mead LE Estes M Prater DN Derr-Yellin E. *In vitro* hyperglycemia or a diabetic intrauterine environment reduces neonatal endothelial colony-forming cell numbers and function. Diabetes (2008) 57:724–31. 10.2337/db07-1507 18086900

[59] Blue EK Sheehan BM Nuss ZV Boyle FA Hocutt CM Gohn CR. Epigenetic regulation of placenta-specific 8 contributes to altered function of endothelial colony-forming cells exposed to intrauterine gestational diabetes mellitus. Diabetes (2015) 64:2664–75. 10.2337/db14-1709 25720387 PMC4477353

[60] Leicht SF Schwarz TM Hermann PC Seissler J Aicher A Heeschen C. Adiponectin pretreatment counteracts the detrimental effect of a diabetic environment on endothelial progenitors. Diabetes (2011) 60:652–61. 10.2337/db10-0240 21270275 PMC3028367

[61] Au P Daheron LM Duda DG Cohen KS Tyrrell JA Lanning RM. Differential *in vivo* potential of endothelial progenitor cells from human umbilical cord blood and adult peripheral blood to form functional long-lasting vessels. Blood (2008) 111:1302–5. 10.1182/blood-2007-06-094318 17993613 PMC2214740

[62] Yoder MC Mead LE Prater D Krier TR Mroueh KN Li F. Redefining endothelial progenitor cells via clonal analysis and hematopoietic stem/progenitor cell principals. Blood (2007) 109:1801–9. 10.1182/blood-2006-08-043471 17053059 PMC1801067

[63] Javed MJ Mead LE Prater D Bessler WK Foster D Case J. Endothelial colony forming cells and mesenchymal stem cells are enriched at different gestational ages in human umbilical cord blood. Pediatr Res. (2008) 64:68–73. 10.1203/PDR.0b013e31817445e9 18360305

[64] Ligi Simoncini S Tellier E Vassallo PF Sabatier F Guillet B. A switch toward angiostatic gene expression impairs the angiogenic properties of endothelial progenitor cells in low birth weight preterm infants. Blood (2011) 118:1699–709. 10.1182/blood-2010-12-325142 21659549

[65] Vassallo PF Simoncini S Ligi I Chateau AL Bachelier R Robert S. Accelerated senescence of cord blood endothelial progenitor cells in premature neonates is driven by SIRT1 decreased expression. Blood (2014) 123:2116–26. 10.1182/blood-2013-02-484956 24518759

[66] Simoncini S Chateau AL Robert S Todorova D Yzydorzick C Lacroix R. Biogenesis of pro-senescent microparticles by endothelial colony forming cells from premature neonates is driven by SIRT1-dependent epigenetic regulation of MKK6. Sci Rep. (2017) 7:8277. 10.1038/s41598-017-08883-1 28811647 PMC5557933

[67] Moreno-Luna R Munoz-Hernandez R Lin RZ Miranda ML Vallejo-Vaz AJ Stiefel P. Maternal body-mass index and cord blood circulating endothelial colony-forming cells. J Pediatr. (2014) 164:566–71. 10.1016/j.jpeds.2013.10.063 24315508 PMC3943964

[68] Munoz-Hernandez R Miranda ML Stiefel P Lin RZ Praena-Fernandez JM Dominguez-Simeon MJ. Decreased level of cord blood circulating endothelial colony-forming cells in preeclampsia. Hypertension (Dallas, Tex. : 1979) (2014) 64:165–71. 10.1161/HYPERTENSIONAHA.113.03058 24752434 PMC4057301

[69] Baker CD Ryan SL Ingram DA Seedorf GJ Abman SH Balasubramaniam V. Endothelial colony-forming cells from preterm infants are increased and more susceptible to hyperoxia. Am J Respir Crit Care Med. (2009) 180:454–61. 10.1164/rccm.200901-0115OC 19483112 PMC2742761

[70] Fujinaga H Baker CD Ryan SL Markham NE Seedorf GJ Balasubramaniam V. Hyperoxia disrupts vascular endothelial growth factor-nitric oxide signaling and decreases growth of endothelial colony-forming cells from preterm infants. Am J Physiol Lung Cell Mol Physiol. (2009) 297:L1160–9. 10.1152/ajplung.00234.2009 19734318 PMC2793187

[71] Baker CD Balasubramaniam V Mourani PM Sontag MK Black CP Ryan SL. Cord blood angiogenic progenitor cells are decreased in bronchopulmonary dysplasia. Eur Respir J. (2012) 40:1516–22. 10.1183/09031936.00017312 22496315 PMC5596882

[72] Borghesi Massa M Campanelli R Bollani L Tzialla C Figar TA. Circulating endothelial progenitor cells in preterm infants with bronchopulmonary dysplasia. Am J Respir Crit Care Med. (2009) 180:540–6. 10.1164/rccm.200812-1949OC 19574444

[73] Critser PJ Yoder MC. Endothelial colony-forming cell role in neoangiogenesis and tissue repair. Curr Opin Organ Trans. (2010) 15:68–72. 10.1097/MOT.0b013e32833454b5 19898235 PMC2880951

[74] Tasev D Koolwijk P van Hinsbergh VW. Therapeutic potential of human-derived endothelial colony-forming cells in animal models. Tissue Eng Part B Rev. (2016) 22:371–82. 10.1089/ten.teb.2016.0050 27032435

[75] Melero-Martin JM Khan ZA Picard A Wu X Paruchuri S Bischoff J. *In vivo* vasculogenic potential of human blood-derived endothelial progenitor cells. Blood (2007) 109:4761–8. 10.1182/blood-2006-12-062471 17327403

[76] Baker CD Seedorf GJ Wisniewski BL Black CP Ryan SL Balasubramaniam V. Endothelial colony-forming cell conditioned media promote angiogenesis *in vitro* and prevent pulmonary hypertension in experimental bronchopulmonary dysplasia. Am J Physiol Lung Cell Mol Physiol. (2013) 305:L73–81. 10.1152/ajplung.00400.2012 23666751 PMC3726941

[77] Burger D Vinas JL Akbari S Dehak H Knoll W Gutsol A. Human endothelial colony-forming cells protect against acute kidney injury: role of exosomes. Am J Pathol. (2015) 185:2309–23. 10.1016/j.ajpath.2015.04.010 26073035

[78] Collett JA Mehrotra P Crone A Shelley WC Yoder MC Basile DP. Endothelial colony-forming cells ameliorate endothelial dysfunction via secreted factors following ischemia-reperfusion injury. Am J Physiol. Renal Physiol. (2017) 312:F897–907. 10.1152/ajprenal.00643.2016 28228404 PMC5451554

[79] Vinas JL Burger D Zimpelmann J Haneef R Knoll W Campbell P. Transfer of microRNA-486-5p from human endothelial colony forming cell-derived exosomes reduces ischemic kidney injury. Kidney Int. (2016) 90:1238–50. 10.1016/j.kint.2016.07.015 27650731

[80] Au P Tam J Fukumura D Jain RK. Bone marrow-derived mesenchymal stem cells facilitate engineering of long-lasting functional vasculature. Blood (2008) 111:4551–8. 10.1182/blood-2007-10-118273 18256324 PMC2343592

[81] Melero-Martin JM De Obaldia ME Kang SY Khan ZA Yuan L Oettgen P. Engineering robust and functional vascular networks *in vivo* with human adult and cord blood-derived progenitor cells. Circ Res. (2008) 103:194–202. 10.1161/CIRCRESAHA.108.178590 18556575 PMC2746761

[82] Lin RZ Moreno-Luna R Li D Jaminet SC Greene AK Melero-Martin JM. Human endothelial colony-forming cells serve as trophic mediators for mesenchymal stem cell engraftment via paracrine signaling. Proc Natl Acad Sci USA. (2014) 111:10137–42. 10.1073/pnas.1405388111 24982174 PMC4104912

[83] Traktuev DO Prater DN Merfeld-Clauss S Sanjeevaiah AR Saadatzadeh MR Murphy M. Robust functional vascular network formation *in vivo* by cooperation of adipose progenitor and endothelial cells. Circ Res. (2009) 104:1410–20. 10.1161/CIRCRESAHA.108.190926 19443841

[84] Kang KT Lin RZ Kuppermann D Melero-Martin JM Bischoff J. Endothelial colony forming cells and mesenchymal progenitor cells form blood vessels and increase blood flow in ischemic muscle. Sci Rep. (2017) 7:770. 10.1038/s41598-017-00809-1 28396600 PMC5429692

[85] Moubarik C Guillet B Youssef B Codaccioni JL Piercecchi MD Sabatier F. Transplanted late outgrowth endothelial progenitor cells as cell therapy product for stroke. Stem Cell Rev. (2011) 7:208–20. 10.1007/s12015-010-9157-y 20526754

[86] Schwarz TM Leicht SF Radic T Rodriguez-Arabaolaza I Hermann PC Berger F. Vascular incorporation of endothelial colony-forming cells is essential for functional recovery of murine ischemic tissue following cell therapy. Arterioscler Thrombo Vasc Biol. (2012) 32:e13–21. 10.1161/ATVBAHA.111.239822 22199368

[87] Bouvard C Gafsou B Dizier B Galy-Fauroux I Lokajczyk A Boisson-Vidal C. alpha6-integrin subunit plays a major role in the proangiogenic properties of endothelial progenitor cells. Arterioscler Thrombo Vasc Biol. (2010) 30:1569–75. 10.1161/ATVBAHA.110.209163 20508204

[88] Saif J Schwarz TM Chau DY Henstock J Sami P Leicht SF. Combination of injectable multiple growth factor-releasing scaffolds and cell therapy as an advanced modality to enhance tissue neovascularization. Arterioscler Thromb Vasc Biol. (2010)30:1897–904. 10.1161/ATVBAHA.110.207928 20689075

[89] Palii CG Vulesevic B Fraineau S Pranckeviciene E Griffith AJ Chu A. Trichostatin A enhances vascular repair by injected human endothelial progenitors through increasing the expression of TAL1-dependent genes. Cell Stem Cell (2014) 14:644–57. 10.1016/j.stem.2014.03.003 24792117

[90] Smadja DM Bieche I Silvestre JS Germain S Cornet A Laurendeau I. Bone morphogenetic proteins 2 and 4 are selectively expressed by late outgrowth endothelial progenitor cells and promote neoangiogenesis. Arterioscler Thromb Vasc Biol. (2008) 28:2137–43. 10.1161/ATVBAHA.108.168815 18818419

[91] Lee SH Lee JH Asahara T Kim YS Jeong HC Ahn Y. Genistein promotes endothelial colony-forming cell (ECFC) bioactivities and cardiac regeneration in myocardial infarction. PLoS ONE (2014) 9:e96155. 10.1371/journal.pone.0096155 24830850 PMC4022670

[92] Ding J Zhao Z Wang C Wang CX Li PC Qian C. Bioluminescence imaging of transplanted human endothelial colony-forming cells in an ischemic mouse model. Brain Res. (2016) 1642:209–18. 10.1016/j.brainres.2016.03.045 27038754

[93] Zhang Y Li Y Wang S Han Z Huang X Li S. Transplantation of expanded endothelial colony-forming cells improved outcomes of traumatic brain injury in a mouse model. J Surg Res. (2013) 185:441–9. 10.1016/j.jss.2013.05.073 23953790

[94] Huang XT Zhang YQ Li SJ Li SH Tang Q Wang ZT. Intracerebroventricular transplantation of *ex vivo* expanded endothelial colony-forming cells restores blood-brain barrier integrity and promotes angiogenesis of mice with traumatic brain injury. J Neurotrauma (2013) 30:2080–8. 10.1089/neu.2013.2996 23957220 PMC3868401

[95] Medina RJ O'Neill CL Humphreys MW Gardiner TA Stitt AW. Outgrowth endothelial cells: characterization and their potential for reversing ischemic retinopathy. Invest Ophthalmol Visual Sci. (2010) 51:5906–13. 10.1167/iovs.09-4951 20554606

[96] Dellett M Brown ED Guduric-Fuchs J O'Connor A Stitt AW Medina RJ. MicroRNA-containing extracellular vesicles released from endothelial colony-forming cells modulate angiogenesis during ischaemic retinopathy. J Cell Mol Med. (2017) 21:3405–19. 10.1111/jcmm.13251 28631889 PMC5706503

[97] Reid E Guduric-Fuchs J O'Neill CL Allen LD S. Chambers EJ Stitt AW. Preclinical evaluation and optimization of a cell therapy using human cord blood-derived endothelial colony-forming cells for ischemic retinopathies. Stem Cells Transl Med. (2018) 7:59–67. 10.1002/sctm.17-0187 29164803 PMC5746158

[98] Alphonse RS Vadivel A Fung M Shelley WC Critser PJ Ionescu L e tal. Existence, functional impairment, and lung repair potential of endothelial colony-forming cells in oxygen-induced arrested alveolar growth. Circulation (2014) 129:2144–57. 10.1161/CIRCULATIONAHA.114.009124 24710033 PMC4162516

[99] Basile DP Collett JA Yoder MC. Endothelial colony-forming cells and pro-angiogenic cells: clarifying definitions and their potential role in mitigating acute kidney injury. Acta Physiol. (2018) 222. [Epub ahead of print]. 10.1111/apha.12914 28656611 PMC5745310

[100] Banno K Yoder MC. Tissue regeneration using endothelial colony-forming cells: promising cells for vascular repair. Pediatric Res. (2018) 83:283–90. 10.1038/pr.2017.231 28915234

[101] Matsui H Shibata M Brown B Labelle A Hegadorn C Andrews C. Ex vivo gene therapy for hemophilia A that enhances safe delivery and sustained *in vivo* factor VIII expression from lentivirally engineered endothelial progenitors. Stem Cells (Dayton, Ohio) (2007) 25:2660–9. 10.1634/stemcells.2006-0699 17615271

[102] Lin Y Chang L Solovey A Healey JF Lollar P Hebbel RP. Use of blood outgrowth endothelial cells for gene therapy for hemophilia A. Blood (2002)99:457–62. 10.1182/blood.V99.2.457 11781225

[103] Ozelo MC Vidal B Brown C Notley C Hegadorn C Webster S. Omental implantation of BOECs in hemophilia dogs results in circulating FVIII antigen and a complex immune response. Blood (2014) 123:4045–53. 10.1182/blood-2013-12-545780 24829206

[104] De Meyer SF Vanhoorelbeke K Chuah MK Pareyn I Gillijns V Hebbel RP. Phenotypic correction of von Willebrand disease type 3 blood-derived endothelial cells with lentiviral vectors expressing von Willebrand factor. Blood (2006) 107:4728–36. 10.1182/blood-2005-09-3605 16478886 PMC1895808

[105] Lin RZ Dreyzin A Aamodt K Li D Jaminet SC Dudley AC. Induction of erythropoiesis using human vascular networks genetically engineered for controlled erythropoietin release. Blood (2011) 118:5420–8. 10.1182/blood-2011-08-372946 21937702 PMC3217346

[106] Dudek AZ Bodempudi V Welsh BW Jasinski P Griffin RJ Milbauer L. Systemic inhibition of tumour angiogenesis by endothelial cell-based gene therapy. Br J Cancer (2007) 97:513–22. 10.1038/sj.bjc.6603883 17653078 PMC2360342

[107] Bodempudi V Ohlfest JR Terai K Zamora EA Vogel RI Gupta K. Blood outgrowth endothelial cell-based systemic delivery of antiangiogenic gene therapy for solid tumors. Cancer Gene Ther. (2010)17:855–63. 10.1038/cgt.2010.42 20725100 PMC4944849

[108] Wei J Wahl J Nakamura T Stiller D Mertens T Debatin KM. Targeted release of oncolytic measles virus by blood outgrowth endothelial cells *in situ* inhibits orthotopic gliomas. Gene Ther. (2007)14:1573–86. 10.1038/sj.gt.3303027 17898797

[109] Critser PJ Kreger ST Voytik-Harbin SL Yoder MC. Collagen matrix physical properties modulate endothelial colony forming cell-derived vessels *in vivo*. Microvas Res. (2010) 80:23–30. 10.1016/j.mvr.2010.03.001 20219180 PMC2880164

[110] Kim PH Yim HG Choi YJ Kang BJ Kim J Kwon SM. Injectable multifunctional microgel encapsulating outgrowth endothelial cells and growth factors for enhanced neovascularization. J Control Release Soc. (2014) 187:1–13. 10.1016/j.jconrel.2014.05.010 24852096

[111] Allen P Kang KT Bischoff J. Rapid onset of perfused blood vessels after implantation of ECFCs and MPCs in collagen, PuraMatrix and fibrin provisional matrices. J Tissue Eng Regen Med. (2015) 9:632–6. 10.1002/term.1803 23955835

[112] Chen X Aledia AS Popson SA Him L Hughes CC George SC. Rapid anastomosis of endothelial progenitor cell-derived vessels with host vasculature is promoted by a high density of cotransplanted fibroblasts. Tissue Eng Part A (2010) 16:585–94. 10.1089/ten.tea.2009.0491 19737050 PMC2813071

[113] Liu Y Teoh SH Chong MS Lee ES Mattar CN Randhawa NK. Vasculogenic and osteogenesis-enhancing potential of human umbilical cord blood endothelial colony-forming cells. Stem Cells (Dayton, Ohio) (2012) 30:1911–24. 10.1002/stem.1164 22761003

[114] Levengood SK Poellmann MJ Clark SG Ingram DA Yoder MC Johnson AJ. Human endothelial colony forming cells undergo vasculogenesis within biphasic calcium phosphate bone tissue engineering constructs. Acta Biomater. (2011) 7:4222–8. 10.1016/j.actbio.2011.07.006 21798379

[115] He J Decaris ML Leach JK. Bioceramic-mediated trophic factor secretion by mesenchymal stem cells enhances *in vitro* endothelial cell persistence and *in vivo* angiogenesis. Tissue Eng Part A (2012) 18:1520–8. 10.1089/ten.tea.2011.0127 22546052 PMC3397122

[116] Fuchs S Ghanaati S Orth C Barbeck M Kolbe M Hofmann A. Contribution of outgrowth endothelial cells from human peripheral blood on *in vivo* vascularization of bone tissue engineered constructs based on starch polycaprolactone scaffolds. Biomaterials (2009) 30:526–34. 10.1016/j.biomaterials.2008.09.058 18977026

[117] Denecke B Horsch LD Radtke S Fischer JC Horn PA Giebel B. Human endothelial colony-forming cells expanded with an improved protocol are a useful endothelial cell source for scaffold-based tissue engineering. J Tissue Eng Regenerat Med. (2015) 9:E84–97. 10.1002/term.1673 23436759

[118] Kim KL Han DK Park K Song SH Kim JY Kim JM. Enhanced dermal wound neovascularization by targeted delivery ofendothelial progenitor cells using an RGD-g-PLLA scaffold. Biomaterials (2009) 30:3742–8. 10.1016/j.biomaterials.2009.03.053 19394079

[119] Kung EF Wang F Schechner JS. *In vivo* perfusion of human skin substitutes with microvessels formed by adult circulating endothelial progenitor cells. Dermatol Surg. (2008) 34:137–46. 10.1111/j.1524-4725.2007.34030.x 18190540

[120] Hendrickx B Verdonck K Van den Berge S Dickens S Eriksson E Vranckx JJ. Integration of blood outgrowth endothelial cells in dermal fibroblast sheets promotes full thickness wound healing. Stem Cells (Dayton, Ohio) (2010) 28:1165–77. 10.1002/stem.445 20506500

[121] Fraineau S Palii CG McNeill B Ritso M Shelley WC Prasain N. Epigenetic activation of pro-angiogenic signaling pathways in human endothelial progenitors increases vasculogenesis. Stem Cell Rep. (2017) 9:1573–87. 10.1016/j.stemcr.2017.09.009 29033304 PMC5830028

[122] Fraineau S Palii CG Allan DS Brand M. Epigenetic regulation of endothelial-cell-mediated vascular repair. FEBS J. (2015) 282:1605–29. 10.1111/febs.13183 25546332

[123] Bennis Y Sarlon-Bartoli G Guillet B Lucas L Pellegrini L Velly L. Priming of late endothelial progenitor cells with erythropoietin before transplantation requires the CD131 receptor subunit and enhances their angiogenic potential. J Thrombo Haemost. (2012) 10:1914–28. 10.1111/j.1538-7836.2012.04835.x 22738133

[124] Lee JH Lee SH Choi SH Asahara T Kwon SM. The sulfated polysaccharide fucoidan rescues senescence of endothelial colony-forming cells for ischemic repair. Stem Cells (Dayton, Ohio) (2015) 33:1939–51. 10.1002/stem.1973 25693733

[125] Kim H Prasain N Vemula S Ferkowicz MJ Yoshimoto M Voytik-Harbin SL. Human platelet lysate improves human cord blood derived ECFC survival and vasculogenesis in three dimensional (3D) collagen matrices. Microvasc Res. (2015) 101:72–81. 10.1016/j.mvr.2015.06.006 26122935 PMC4537830

[126] Green LA Njoku V Mund J Case J Yoder M Murphy MP. Endogenous transmembrane TNF-alpha protects against premature senescence in endothelial colony forming cells. Circ Res. (2016) 118:1512–24. 10.1161/CIRCRESAHA.116.308332 27076598 PMC4867129

[127] Wakabayashi T Naito H Suehiro JI Lin Y Kawaji H Iba T. CD157 marks tissue-resident endothelial stem cells with homeostatic and regenerative properties. Cell Stem Cell (2018) 22:384–97.e6. 10.1016/j.stem.2018.01.010 29429943

[128] Tasev D van Wijhe MH Weijers EM van Hinsbergh VW Koolwijk P. Long-term expansion in platelet lysate increases growth of peripheral blood-derived endothelial-colony forming cells and their growth factor-induced sprouting capacity. PLoS ONE (2015) 10:e0129935. 10.1371/journal.pone.0129935 26076450 PMC4468160

[129] Siegel G Fleck E Elser S Hermanutz-Klein U Waidmann M Northoff H. Manufacture of endothelial colony-forming progenitor cells from steady-state peripheral blood leukapheresis using pooled human platelet lysate. Transfusion (2018) 58:1132–42. 10.1111/trf.14541 29473177

[130] Lapidos KA Sprague SM Ameer GA. Impact of serum source and inflammatory cytokines on the isolation of endothelial colony-forming cells from peripheral blood. J Tissue Eng Regen Med. (2014) 8:747–56. 10.1002/term.1580 22888041

[131] Lin RZ Moreno-Luna R Munoz-Hernandez R Li D Jaminet SC Greene AK. Human white adipose tissue vasculature contains endothelial colony-forming cells with robust *in vivo* vasculogenic potential. Angiogenesis (2013) 16:735–44. 10.1007/s10456-013-9350-0 23636611 PMC3762916

